# The Effect of the Pyrolysis Temperature of a Leather–Textile Mixture from Post-Consumer Footwear on the Composition and Structure of Carbonised Materials

**DOI:** 10.3390/ma17225649

**Published:** 2024-11-19

**Authors:** Anna Kowalik-Klimczak, Monika Łożyńska, Maciej Życki, Bogusław Woźniak

**Affiliations:** Łukasiewicz Research Network–Institute for Sustainable Technology, Pułaskiego St. 6/10, 26-600 Radom, Poland; monika.lozynska@itee.lukasiewicz.gov.pl (M.Ł.); maciej.zycki@itee.lukasiewicz.gov.pl (M.Ż.); boguslaw.wozniak@itee.lukasiewicz.gov.pl (B.W.)

**Keywords:** pyrolysis, post-consumer footwear, mixture of leather and textiles, composition and structure of carbonised materials, valorisation of waste

## Abstract

This paper presents an investigation into the use of pyrolysis to valorise solid waste in the form of post-consumer footwear uppers. A heterogenous leather and textile mixture is studied, produced by crushing some representative samples of post-consumer footwear uppers. The waste has a low ash content and a high net calorific value, which translates into the high gross calorific value of the material. In addition, it contains relatively little S and Cl, which is promising for its use in the process of pyrolysis. The effect of the pyrolysis temperature on the efficiency of carbonising leather and textile mixtures, their physico-chemical parameters, elemental composition, and structure, as well as the development of a specific surface, is investigated. The research results imply that as the pyrolysis temperature grows, the carbonisation efficiency declines. The produced materials consist primarily of C, O, N, and H, whose contents depend on the pyrolysis temperature. Moreover, all the carbonised materials display the presence of two G and D bands, which is typical for carbon materials. Based on the peak intensities of the bands, I_D_/I_G_ coefficients are calculated to assess the organisation of the materials’ structures. As the pyrolysis temperature rises, the structural organisation declines, contributing to an increased material porosity and, thus, a greater specific surface of the carbonised materials. This study contributes data on the thermal management and pyrolysis of leather and textile waste into useful carbonised materials. Investigating the applicability of carbonised materials is projected as the next stage of research work.

## 1. Introduction

The footwear industry plays an important role in the global economy, providing necessary protective foot products and acting as part of an individual’s sense of fashion and style. Manufacturing in this sector consists of multiple stages, including the design and choice of materials, production, distribution, and retail. Sustainable material selection, limited use of toxic substances, and prevention and recycling of production waste are now among the key factors underlying the footwear manufacturing process, and they help greatly restrict factors that are degrading the environment [1,2].

The dynamic development of the footwear industry resulted in ca. 24 billion pairs of footwear being manufactured worldwide in 2022. The rapid production and consumption of footwear, in accordance with the linear economic model, is responsible for the considerable quantities of post-consumer footwear stored in landfills [3]. The multi-material composition of this waste is an issue when selecting rational methods for its management/disposal. Natural leather, textiles (cotton and polyester), and foam (polyurethane and polyethene) are most commonly used to make uppers. In turn, soles are most often made of soft polyvinyl chloride, polyurethanes, thermoplastic rubber, and vulcanised rubber. The presence of multiple metal elements (eyelets, buckles, zippers, and structural parts) and the application of dedicated glues that allow for lasting connections of footwear elements are not without significance. Such a complex material composition of footwear requires technological and design solutions that enable the disassembly, crushing, and splitting of the particular fractions to then make some new elements out of them [4].

Mechanical recycling (grinding and adding to new raw material) is most often suggested as the way to manage post-consumer footwear soles since it allows for the recovery of components to manufacture new soles [5], laminates [6], composite materials [7], and epoxy coatings [8]. Research into the thermal processing of natural leathers and textiles suggests, in turn, the option of valorising shoe uppers in the process of pyrolysis [9,10]. The state-of-the art of the different approaches available in the scientific literature has shown that appropriately guided pyrolysis allows for the processing of leather waste [11,12], tanning sludge [13,14], and animal hair [15,16] into carbonised materials with wide applicability [17]. Carbon materials based on tanning waste may serve to remove organic compounds from aqueous solutions, especially pigments [12,13,14] and pharmaceuticals [16], and purify biogas [18], fertilise soil [19], and store energy in lithium-ion batteries [20].

The rapidly changing fashion trends generate huge amounts of textile waste globally [21]. Textile waste chiefly consists of natural, synthetic, organic, and inorganic fibres. The heterogeneity and complexity of textile waste make recycling difficult in economic terms. Pyrolysis, which transforms heterogeneous and complex waste into added-value products, is therefore suggested for this purpose [22]. Textile waste materials can be pyrolysed into active carbon precursors that, if properly modified chemically, exhibit high sorption capabilities with regard to heavy metals [23], pigments [24,25], and pharmaceuticals [26,27].

The possibility of using pyrolysis to valorise post-consumer footwear uppers, which comprise a mixture of leather and textile materials, has been analysed in this paper. The effect of the pyrolysis temperature on the physico-chemical properties, composition, structure, and formation of specific surfaces of carbonised materials produced by means of thermal treatment of uppers without oxygen access to the reaction chamber was investigated.

## 2. Materials and Methods

### 2.1. The Physico-Chemical Parameters of Waste and Carbonised Materials

Feedstock in the form of some representative samples of crushed post-consumer footwear uppers, i.e., mixtures of leather and textile materials, was studied. An ML-SC10 mechanical crusher (Ming Lee Industrial (HK) Limited, Hong Kong, China) with a power of 4.5 kW and six elements with vertically mounted knives was used for waste pre-treatment. The operation was continuous, with each piece of waste being sequentially fed into the crusher. The grinding time of a single piece of waste was from 20 to 30 s at room temperature, with the machine’s rotation speed maintained at 650 RPM. An 8 mm sieve was installed at the machine’s outlet to limit the particle size. The waste material prepared using this method was subsequently subjected to pyrolysis.

The waste leather–textile materials before and after the pyrolysis were described in terms of moisture content, dry mass, ash, and calorific value. The moisture and dry mass contents were used in the weighing method and by drying the samples at 105 °C [28]. The ash content was assayed, and the weighing method was used to roast the samples at 815 °C [29]. A binder laboratory drier, a Thermo Scientific (Waltham, MA, USA) muff oven, and Ohaus (Nänikon, Switzerland) analytical scales were utilised. The calorific values were determined by means of an IKA calorimeter (Warsaw, Poland) equipped with software for calculating net calorific value [30].

### 2.2. The Process of Pyrolysis

Pyrolysis was conducted using an FCF 2R retort furnace manufactured by Czylok (Jastrzębie-Zdrój, Poland), using CO_2_ flows of 2 dm^3^/min at temperature range of 500–800 °C. For each temperature setting, the process was repeated three times to determine the mean productivity of carbonised material. To optimise the process, the temperature of the water jacket surrounding the furnace hatch was regulated with a thermostat to mitigate fluctuations across different retort zones. A three-step temperature increase protocol was implemented, and the cycle lasted for 135 min. In the initial stage, the leather–textile mixture underwent preliminary thermal treatment for the first 40 min, during which the temperature was uniformly increased for all pyrolysis variants, i.e., from 20 °C to 200 °C at a rate of 5 °C/min. Further heating was conducted under four thermally distinct conditions: to a temperature 50 °C below the target temperature for 40 min, followed by stabilisation for 10 min and then elevation to the final temperatures of 500, 600, 700, and 800 °C for 15 min, with an isothermal state maintained for 30 min. The employment of a three-step temperature increase procedure aimed to minimise the effect of thermal inertia, which has a significant impact on the process and the products obtained. At the end of the process, samples were retained in the furnace retort under a CO_2_ atmosphere for 24 h.

The carbonised material samples were then weighed, and the productivity was calculated as follows:

Carbonised material yield = carbonised material mass [g]/initial mass of crushed waste [g] × 100 [%]

A Testchem LMWs vibration mill, including a tungsten carbide grinding vessel, was used to grind and mix the carbonised material samples.

### 2.3. Elemental Composition and Identification of Functional Groups

A Vario Micro Cube elemental analyser manufactured by Elementar (Langenselbold, Germany) served to analyse the elemental composition of leather–textile mixtures and carbonised materials. The initial content of carbon (C), hydrogen (H), nitrogen (N), and sulphur (S) were assayed via elemental microanalysis using a thermal-conductance detector (TCD). The percentage of chlorine (Cl) content was assayed using elemental microanalysis and electrochemical detection (ECD). The results were recalculated for dry samples.

Subsequently, the total metal content in the samples was determined following their prior mineralisation in a mixture of 65% HNO_3_ and 30% H_2_O_2_. The mineralisation process was performed using a MARS 5 digestion oven manufactured by CEM Corporation (Matthews, NC, USA) with a closed-system vessel. Then, the filtered solutions were analysed using a Thermo Scientific iCE 3000 Series atomic absorption spectrometer with flame atomisation (Waltham, MA, USA). The detection limits for the tested metals were as follows: calcium (Ca)—0.7 mg/kg; chromium (Cr)—2.5 mg/kg; iron (Fe)—3.2 mg/kg; potassium (K)—1.8 mg/kg; magnesium (Mg)—2.9 mg/kg; sodium (Na)—1.1 mg/kg; zinc (Zn)—1.5 mg/kg.

In order to identify functional groups present on the carbonised material surface, Fourier-transform infrared spectroscopy (FTIR) was applied. The spectra were recorded with a Jasco FT/IR 6200 spectrometer (Tokyo, Japan) provided with a TGS detector within the spectral range 4000–650 cm^−1^ and a resolution of 4 cm^−1^. The spectrometer was equipped with a PIKE Technology MIRacle single reflection ATR accessory containing a plate for powdered samples (contact area: ϕ 1.8 mm) and a high-pressure clamp (10,141 psi upon a sample). The carbonised sample was evenly distributed on the diamond crystal in the form of a compact layer.

### 2.4. Morphology, Structure, and Surface Formation

The morphology of leather–textile mixtures and carbonised materials was investigated by means of a Hitachi scanning electron microscope (Krefeld, Germany) equipped with an EDS X-ray microanalyser from Thermo Scientific (Waltham, MA, USA). To perform the analysis, a carbonised sample was attached to a piece of adhesive tape.

Meanwhile, the structure of the carbonised materials produced was tested using a Raman Jasco NRS-5100 spectroscope (Tokyo, Japan). Raman spectra were produced at room temperature by applying laser excitation at a wavelength of 532 nm and exposure time of 120 s. The spectra were recorded for wavenumbers from 300 to 3200 cm^−1^ and a resolution of 3.62 cm^−1^. The carbonised samples were evenly spread on a microscope slide in the form of a compact layer, then placed on an automatic table, and the measurement point was determined using a built-in high-resolution CMOS camera (Olympus, Tokyo, Japan).

The formation of the carbonised material surface was assessed on the basis of N_2_ (77 K) adsorption isotherms plotted with a Quantachrome AUTOSORB IQ analyser (Anton Paar QuantaTec, Graz, Austria). The samples were first vacuum degassed (10^−7^ bar) at 350 °C for 12 h. Using data from the physisorption process, specific surfaces, pore distribution, and volumes were calculated. The specific surface was determined by means of Brunauer–Emmett–Teller (MBET) multiple-point method. The pore volume was computed using the quenched solid density functional theory (QSDFT) to characterise micro- and mesopores. The t-plot method serves to calculate the micropores’ volume and surface.

## 3. Results and Discussion

### 3.1. Pyrolysis of Waste Leather–Textile Mixtures

**Figure 1:**
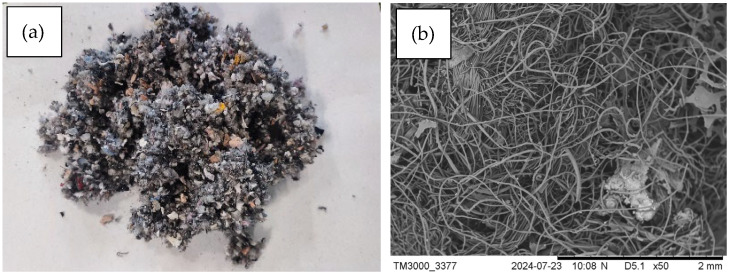
Leather and textile mixture waste materials: a photo (**a**) and SEM microphotograph (**b**).

The samples of ground leather–textile mixture waste materials were dry and free from any clear impurities (Figure 1). They exhibited low ash contents and high calorific values, which translates into high net calorific values, equivalent to those of solid fuels (Table 1).

The crushed waste leather–textile mixtures were subject to pyrolysis at variable temperatures (500–800 °C). The investigations implied that the pyrolysis produced carbonised materials free from liquid oil fractions. As the pyrolysis temperature grows, the yield of carbonised materials reduces (Table 2).

**Figure 2:**
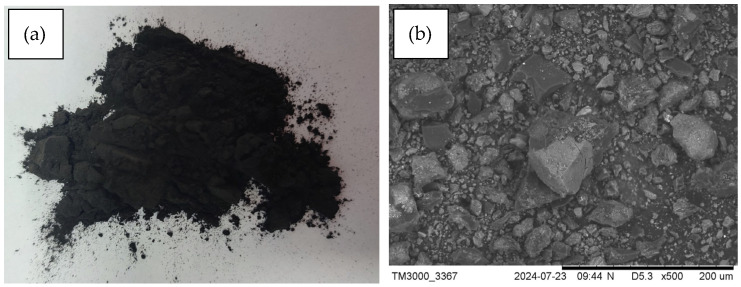
Carbonised material produced by the pyrolysis of waste leather–textile mixtures: a photo (**a**) and a SEM microphotograph (**b**).

The carbonised materials produced by the pyrolysis of waste leather–textile mixtures (designated C500, C600, C700, and C800, respectively, for the pyrolysis temperatures of 500 °C, 600 °C, 700 °C, and 800 °C) are loose (Figure 2a) and display a high dispersion of the particular fraction sizes, a smooth structure, and multiple inclusions of bright pellets visible in SEM (Figure 2b). El-Hout et al. [31] believe this may indicate the formation of stable, highly organised carbon structures, including evenly distributed particle aggregates, most likely in the form of Cr_2_O_3_.

The crushed carbonised materials were described in terms of moisture content, dry mass, ash, gross, and net calorific values (Table 3).

The test results demonstrated some adverse energetic parameters of the carbonised materials produced, in particular, a high ash content and low calorific values. The ash content has a significant effect on the sorption performance of carbon materials. A higher ash content generally reduces the adsorption capacity of organic compounds such as phenol. However, it can increase the adsorption of certain substances, such as fulvic acid and arsenic, due to interactions with metal oxides and ions in the ash [32]. High-ash-content biomass can be a promising precursor for environmentally friendly carbon products, as it requires less alkali consumption during activation and can yield larger surface areas [33].

### 3.2. The Composition and Structure of Carbonised Materials

The elemental compositions of carbonised material made in the pyrolysis of leather–textile mixtures at varied temperatures and investigated with an elemental analyser for the C/H/N/S/Cl assay are listed in Table 4. The results prove the effect of pyrolysis temperature growth on C content rises and reduced H and O contents in the carbonised materials, as well as no dependence between N, S, and Cl contents and the pyrolysis temperature. It is important to note that the contents of S (~0.1% m/m) and Cl (<0.05% m/m) in all the carbonised material types are negligible. The low levels of S and Cl present in the material may be advantageous for its utilisation in pyrolysis, as well as for the potential application of the resulting carbonised materials for sorption. The relatively low S content in the leather–textile mixture intended for pyrolysis is beneficial as it minimises the risk of emissions during the potential energy use of solid and gaseous products. The emission of gases from burning materials containing S can damage trees and other plant life, inhibit plant growth and damage sensitive ecosystems and waterways, and contribute to respiratory diseases and exacerbate existing heart and lung conditions. Conversely, a low Cl content in the leather–textile mixture intended for the pyrolysis process is beneficial as it mitigates the risk of corrosion-induced destruction of the pyrolysis reactor, which may lead to its leakage. A review of the literature [34] revealed that the S and Cl content of pyrolysis products is dependent on the raw material and the process temperature. The presence of S and Cl is also undesirable when using carbon material as a sorbent for water contaminants, as it may result in the release of unbound forms of these elements into the purified liquid, causing its secondary contamination.

**Figure 3:**
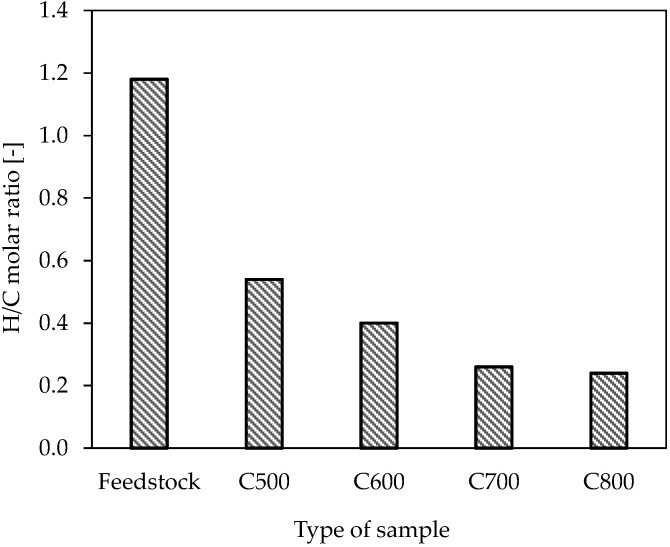
The H/C molar ratio in the waste leather–textile mixtures and the resultant carbonised materials.

The H/C molar ratio in the waste leather–textile mixtures and the resultant carbonised materials are shown in Figure 3. A reduction in the H/C molar ratio with increasing pyrolysis temperature may indicate the dehydration, polymerisation and volatilisation of certain organic structures. Unfortunately, the expected correlation between the O/C molar ratio and the pyrolysis temperature, typical for these phenomena, was not observed [35].

The content of Cr, Zn, Fe, Na, K, Mg, and Ca was determined in leather–textile mixtures and carbonised products derived from them. In the feedstock samples, the highest content observed was for Ca (~32 g/kg), Mg (~1 g/kg), and Zn (~0.8 g/kg). The content levels of the remaining metals were comparable (Cr ~140 mg/kg, Fe ~185 mg/kg, Na ~166 mg/kg, and K ~174 mg/kg). The types of metals and their respective contents in leather–textile mixtures result from the production cycles and chemicals used during the tanning of leather, the processing of fabrics, and the manufacture of footwear uppers. A significant increase in metal content in the carbonised materials in comparison to the textile–leather mixtures was observed. This is due to the decomposition of organic components during pyrolysis, which results in a reduction in the mass of the material [36]. Accordingly, an increase in the metal content in carbonised material was observed (Table 5).

**Figure 4:**
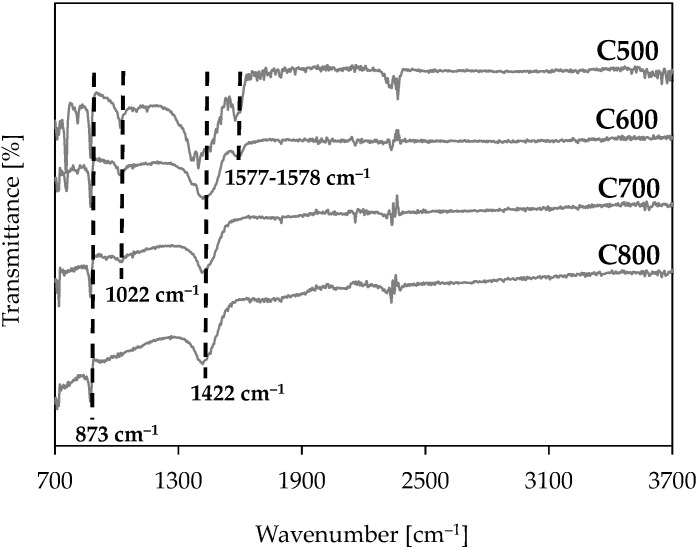
The FTIR spectra of carbonised materials produced by pyrolysis at varied temperatures.

Fourier-transform infrared spectroscopy (FTIR) serves to describe functional groups in the carbonised materials made by the pyrolysis of waste leather–textile mixtures. The FTIR spectra of carbonised materials produced by pyrolysis at varied temperatures in the range of 500–800 °C are illustrated in Figure 4. The band present at the wavelengths of 3600–3100 cm^−1^ (responsible for vibrations stretching the O–H bonds in hydroxy groups) is absent from the samples [31]. The band may be responsible for sample water content, and the lack thereof explains the low moisture content of the carbonised materials (Table 3). Its absence may also be evidence of the lack of O–H bonds, typical for carboxylic acids, alcohols, or phenols [36,37]. Consequently, the 1577–1578 cm^−1^ band, observed for the carbonised materials produced at 500 °C and 600 °C, is attributed to vibrations elongating C=C bonds in the aromatic ring [38,39]. The band vanishes as the temperature rises from 600 °C to 700 °C. This may be caused by the bonds cracking in pyrolysis above 600 °C. The strong peaks at the 1422 cm^−1^ wavenumber, potentially indicative of an aliphatic C-H bond, can be noted in all the samples studied. The band corresponding to the 1022 cm^−1^ wavenumber, responsible for the C-O bond, whose presence may suggest the presence of ring hydrocarbon structures, is observable in carbonised materials made at 500, 600, and 700 °C. The peaks seen for all the carbonised materials at 873 cm^−1^ are characteristic of =C-H group bending vibrations, whose presence may suggest aromatic hydrocarbons [39].

**Figure 5:**
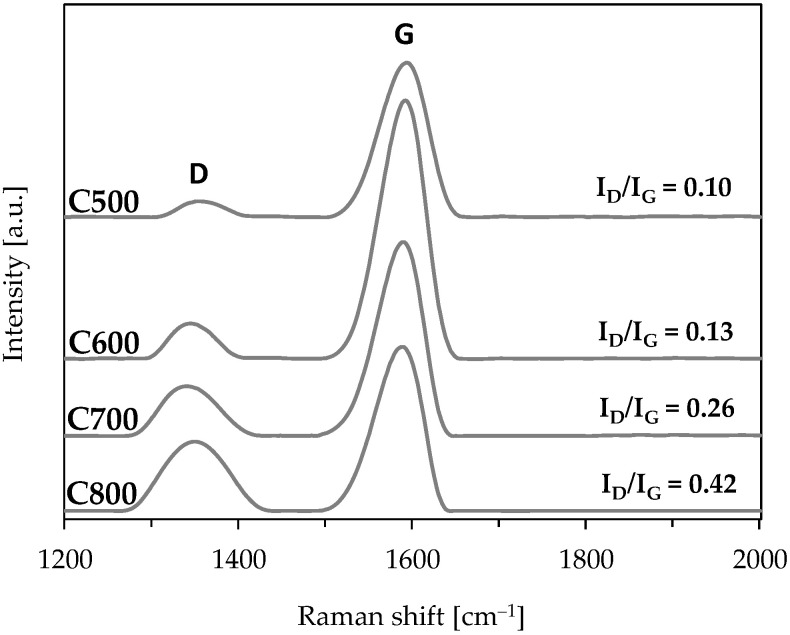
The Raman spectra of carbonised materials made in the pyrolytic processes of leather–textile mixtures at different temperatures.

In turn, the results of Raman spectroscopy testing imply that the pyrolysis temperature has a significant impact on the structure of carbonised materials produced (Figure 5). Two bands typical for carbon materials were found for all the carbonised materials investigated: the G band (defining the graphite structure, due to attribution to in-plane vibrations of sp^2^-bonded carbon) at the wavenumber of approx. 1590 cm^−1^ and D band (the so-called defect band, which arises from the in-plane vibrations of the sp^2^-bonded crystallite carbon) for the wavenumber of ca. 1350 cm^−1^. The individual D and G peaks were relatively symmetrical, repeatable in shape, and clearly separated from each other in all spectra, i.e., they did not overlap. Peak G dominated in all carbonised materials. This means that the share of ordered structures in carbonised materials produced during the pyrolysis processes of leather–textile mixtures carried out in the temperature range of 500–800 °C was higher than that of disordered structures. However, the D peak dominates in carbonised materials made from leather waste [31], while the G peak dominates in those made from textile waste (polyester fibres and, cotton) [40]. The I_D_/I_G_ peak intensity coefficients were determined from the heights of the D and G peaks obtained from the Raman spectra recorded for the carbonised materials produced. I_D_/I_G_ ratios made it possible to assess the organisation of the structure of the obtained materials. The carbonised material produced at 500 °C has the most organised structure. The I_D_/I_G_, in this case, is 0.10. With the increase in the pyrolysis temperature, I_D_/I_G_ increases (it is 0.13, 0.26, and 0.42 for 600, 700, and 800 °C, respectively), and the organisation of carbonised materials’ structure deteriorates. Since the I_D_/I_G_ of commercial graphite is in the range of 0.03–0.17 (as influenced by edge defects, depending on graphite dimensions) [41], the carbonised materials obtained at 500 °C and 600 °C have graphite-like structures. Other researchers obtained a similar correlation between the influence of pyrolysis temperature on the structure of carbonised materials produced from tannery waste [42]. This increasing disorder is a result of different gas species (including CH_4_, CO_2_, and CO) and H_2_O forming throughout pyrolysis. These gasses and H_2_O may evolve throughout the structure of materials, promoting disorder. Graphitisation of the materials occurs only at temperatures above 1000 °C, at which ordered graphite structures have been created [43,44,45].

**Figure 6:**
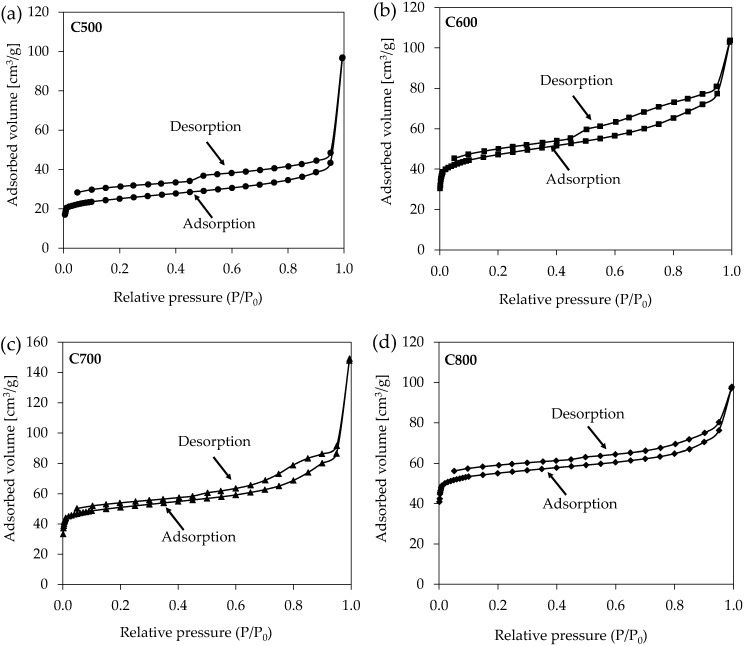
N_2_ adsorption–desorption isotherms of carbonised materials produced through pyrolysis at varying temperatures: 500 °C (**a**), 600 °C (**b**), 700 °C (**c**), and 800 °C (**d**).

The formation of stable, highly organised structures may restrict the porosity of carbon materials and, thus, the formation of specific surface (S_MBET_) [31]. Accordingly, the growth of pyrolysis temperature impaired structure organisation and thus contributed to greater microporosity and S_MBET_. The N_2_ adsorption/desorption isotherms of carbonised materials made by the pyrolysis of waste leather–textile mixtures are shown in Figure 6. A characteristic feature of the obtained isotherms is the occurrence of H4 hysteresis [46]. The adsorption branch in such cases is a composite of types I and II, with a more pronounced uptake at low P/P_0_ associated with micropore filling. The occurrence of such hysteresis is characteristic of the presence of micropores and a large number of mesopores. The carbonised materials produced include micro- and mesopores, whose percentage fraction depends on temperatures applied to the pyrolysis process. The disappearance of H4 hysteresis with the increasing temperature of pyrolysis confirms that the microporosity of the obtained carbon materials increases (Figure 6).

The results presented in Table 6 show that as the pyrolysis temperature increases, the surface area also increases. This phenomenon can be explained by the decomposition of the organic matter at high temperatures, resulting in the formation of new micropores. In addition, the most notable change in porosity of carbonised materials was observed when the temperature was raised from 500 to 600 °C (Table 6).

Testing the application potential of the carbonised materials produced is planned at the next stage of research. However, they are not projected for energetic purposes due to their physico-chemical properties (above all, the high ash content and low net calorific value). The planned work will investigate the option of using the carbonised materials produced at low temperatures (500 °C and 600 °C) to substitute the graphite used to make a variety of products. The carbonised materials produced during low-temperature pyrolysis may be potentially useful in the manufacture of lubricants [47] and regeneration composites [48] for motoring, machine, and defence industries, as well as in the production of modern textiles [49]. In turn, the carbonised materials produced at higher temperatures (700 °C and 800 °C), forming surfaces of approx. 200 m^2^/g, can become sorption material [23,50] and soil fertiliser precursors [19,36]. According to the results of the research conducted by Puchana-Rosero et al. [12], carbonised material produced from tannery sludge has a BET surface area of 491 m^2^/g and is characterised by high adsorption efficiency for simulated effluents (a complex mixture of dyes) at the level of ~94%. Similar research results were obtained by Arcibar-Orozco et al. [13] by transforming chrome-tanned leather shavings into carbon material with a BET surface area of 439 m^2^/g, which can also be used for dye removal, whereas according to Mella et al. [14], even carbonised materials with a very low BET surface area (~1.6 m^2^/g) can remove ~70% of dyes from water through non-covalent or chemical interactions with dyes. However, carbonised material produced through the pyrolysis of animal hair with a BET surface area of 249 m^2^/g [16] has been successfully used to remove amoxicillin and diclofenac from water. Similar results were obtained by Boudrahem et al. [26] using carbonised material produced from textile waste to remove clofibric acid, tetracycline, and paracetamol from water.

The pore size distribution is a crucial factor in the utilisation of carbonised material in agriculture as it directly influences the soil’s capacity to retain water, store nutrients, support microbial activity, and remove pollutants. Therefore, when selecting carbonised material for soil reclamation, it is essential to consider its specific porous properties in relation to the intended recultivation objectives and the type of soil [19]. The results of the research carried out (Table 6) indicate that the structure of carbonised materials produced through the pyrolysis of leather–textile mixtures is completely devoid of macropores, which, by accumulating water in their spaces, increase soil moisture retention for longer periods of time. The carbonised materials produced through the pyrolysis of leather–textile mixtures carried out at a temperature range of 500–700 °C are characterised by a structure dominated by mesopores. Conversely, the carbonised materials produced at a temperature of 800 °C are characterised by a structure in which the micro- and mesopore content is at a similar level. The retention of water in capillary form by carbonised material with micro- and mesopores is less beneficial to plants but has a positive effect on the development of soil microorganisms. The presence of micropores and mesopores provides a habitat for microorganisms, enabling them to utilise organic resources. This leads to an increase in biological activity within the soil and has a beneficial effect on the improvement of its structure and fertility. Furthermore, such carbonised materials demonstrate enhanced sorption capacity of pollutants, i.e., heavy metals, pesticides, and other toxic substances that may be present in the soil. The larger specific surface area facilitates superior binding of pollutants, which limits their mobility and availability for plants and soil microorganisms. According to Skrzypczak et al. [37], sorption properties of carbonised materials produced by pyrolysis of tannery waste make it suitable for its application as a soil conditioner or carrier of microelements for fertilisers. The problem may be the presence of chromium in carbon materials derived from the pyrolysis of chromium tannery waste. The literature data [51] on the pyrolysis of leather waste show that chromium is present in carbonised materials in the highly stable carbide form, which is an inert material, and, as such, is not available to plants. This offers the possibility of using it in agricultural applications, even though it contains chromium.

## 4. Conclusions

The concept of utilising the thermal process to valorise crushed waste post-consumer footwear uppers, a heterogeneous leather–textile mixture, has been verified. The research has proven the possibility of applying pyrolysis to process such waste into useful carbonised materials. The highest carbonised material yield, ca. 23%, could be observed for pyrolysis at 500 °C. The material produced at this temperature exhibits a high degree of organisation, as evidenced by an I_D_/I_G_ of 0.10. The growth in the pyrolysis temperature reduces the productivity of carbonised materials, the organisation of their structure, and the formation of their specific surface. The applicability of carbonised materials is directly related to these parameters. Therefore, carbonised materials will be investigated for future applications in subsequent stages of research. The carbonised materials produced during the pyrolysis of a leather–textile mixture from post-consumer footwear will be examined for their possible use in the sorption of pollutants from water, the fertilisation of soil, and substitute graphite. This research work will contain an analysis of the possible risks related to the impact of carbonised materials on the natural environment.

## Figures and Tables

**Table 1:** Physico-chemical parameters of crushed post-consumer footwear uppers.

Parameter	Value
Moisture content [%]	0.512 ± 0.016
Dry mass [%]	99.488 ± 0.016
Ash content [%] *	6.590 ± 0.270
Gross calorific value [MJ/kg]	24.89 ± 0.18
Net calorific value [MJ/kg]	23.61 ± 0.18

* Dry matter basis.

**Table 2:** The effect of pyrolysis temperature on carbonised material yield.

Pyrolysis Temperature [°C]	Carbonised Material Yield [wt.%]
500	23.0 ± 1.1
600	22.63 ± 0.55
700	21.56 ± 0.46
800	18.12 ± 0.98

**Table 3:** Physico-chemical parameters of carbonised materials produced via pyrolysis at different temperatures.

Parameter	Sample Type			
	C500	C600	C700	C800
Moisture content [%]	0.544 ± 0.005	0.703 ± 0.011	1.610 ± 0.036	4.477 ± 0.074
Dry mass [%]	99.456 ± 0.005	99.297 ± 0.011	98.390 ± 0.036	95.523 ± 0.074
Ash content [%] *	29.520 ± 0.036	31.667 ± 0.079	27.697 ± 0.079	26.52 ± 0.17
Gross calorific value [MJ/kg]	16.56 ± 0.16	16.54 ± 0.13	17.18 ± 0.09	16.38 ± 0.31
Net calorific value [MJ/kg]	16.04 ± 0.16	16.13 ± 0.12	16.86 ± 0.09	16.00 ± 0.31

* Dry matter basis.

**Table 4:** Elemental compositions of carbonised materials produced by pyrolysis at varied temperatures.

Sample Type	Elemental Composition [wt.%]					
	C	H	N	S	Cl	O *
Feedstock	59.39 ± 0.77	5.86 ± 0.22	1.11 ± 0.06	0.013 ± 0.007	0.041 ± 0.008	33.59
C500	50.21 ± 0.14	2.24 ± 0.10	1.79 ± 0.07	0.121 ± 0.004	0.043 ± 0.004	45.59
C600	48.43 ± 0.12	1.62 ± 0.03	1.74 ± 0.01	0.127 ± 0.006	0.049 ± 0.003	48.03
C700	54.43 ± 0.02	1.17 ± 0.04	1.79 ± 0.05	0.101 ± 0.010	0.035 ± 0.003	42.47
C800	55.81 ± 0.08	1.14 ± 0.01	1.76 ± 0.02	0.098 ± 0.009	0.016 ± 0.002	41.18

* Calculated from the difference.

**Table 5:** Content levels of different metals present in carbonised materials are produced through pyrolysis at varying temperatures.

Sample Type	Metal Content [mg/kg] *						
	Cr	Fe	Na	K	Zn	Mg	Ca
C500	396.1 ± 7.7	633.7 ± 5.5	661.2 ± 3.6	322.4 ± 6.7	1591 ± 16	5243 ± 91	107,645 ± 394
C600	433.9 ± 8.7	698 ± 18	660.4 ± 8.0	381 ± 12	1175 ± 68	5468 ± 109	118,912 ± 1639
C700	344.4 ± 5.7	651.9 ± 4.1	730 ± 37	345.0 ± 3.6	727.5 ± 1.2	5185 ± 20	117,191 ± 2859
C800	330.6 ± 2.5	574.4 ± 4.3	608 ± 26	388 ± 10	538.9 ± 2.8	5198 ± 92	100,803 ± 2321

* Wet matter basis.

**Table 6:** The effect of pyrolysis temperature on the formation of specific surface (S_MBET_), micro-, and mesopore volumes and their percentage fraction in carbonised materials made of leather–textile mixtures.

Pyrolysis Temperature [°C]	S_MBET_ [m^2^/g]	Total Pore Volume [cm^2^/g]	Micropore Volume [cm^2^/g]	Mesopore Volume [cm^2^/g]	Fraction of Micropores [%]	Fraction of Mesopores [%]
500	94	0.087	0.017	0.070	19.54	80.46
600	178	0.127	0.046	0.081	36.22	63.78
700	194	0.152	0.045	0.107	29.61	70.39
800	217	0.121	0.065	0.056	53.72	46.28

## Data Availability

Data is contained within the article.
